# *Wolbachia* infection can bias estimates of intralocus sexual conflict

**DOI:** 10.1002/ece3.4744

**Published:** 2018-12-26

**Authors:** Eoin Duffy, C. Ruth Archer, Manmohan Dev Sharma, Monika Prus, Richa A. Joag, Jacek Radwan, Nina Wedell, David J. Hosken

**Affiliations:** ^1^ Institute of Environmental Sciences Jagiellonian University Krakow Poland; ^2^ Science and Engineering Research Support Facility (SERSF) University of Exeter Penryn UK; ^3^ Faculty of Biology Adam Mickiewicz University Poznań Poland

**Keywords:** *Drosophila simulans*, intralocus sexual conflict, sexual antagonism, *Wolbachia*

## Abstract

Males and females share most of their genome and develop many of the same traits. However, each sex frequently has different optimal values for these shared traits, creating intralocus sexual conflict. This conflict has been observed in wild and laboratory populations of insects and affects important evolutionary processes such as sexual selection, the maintenance of genetic variation, and possibly even speciation. Given the broad impacts of intralocus conflict, accurately detecting and measuring it is important. A common way to detect intralocus sexual conflict is to calculate the intersexual genetic correlation for fitness, with negative values suggesting conflict. Here, we highlight a potential confounder of this measure—cytoplasmic incompatibility caused by the intracellular parasite *Wolbachia.* Infection with *Wolbachia* can generate negative intersexual genetic correlations for fitness in insects, suggestive of intralocus sexual conflict*.* This is because cytoplasmic incompatibility reduces the fitness of uninfected females mated to infected males, while uninfected males will not suffer reductions in fitness if they mate with infected females and may even be fitter than infected males. This can lead to strong negative intersexual genetic correlations for fitness, mimicking intralocus conflict. We illustrate this issue using simulations and then present *Drosophila simulans* data that show how reproductive incompatibilities caused by *Wolbachia* infection can generate signals of intralocus sexual conflict. Given that *Wolbachia* infection in insect populations is pervasive, but populations usually contain both infected and uninfected individuals providing scope for cytoplasmic incompatibility, this is an important consideration for sexual conflict research but one which, to date, has been largely underappreciated.

## 1 INTRODUCTION

Sharing an autosomal genome between the sexes generates a pervasive evolutionary problem because traits expressed in both sexes have a common genetic basis, but frequently have different sex‐specific optima (Bonduriansky & Chenoweth, 2009; Mank, Hosken, & Wedell, 2014; Pennell & Morrow, 2013). Accordingly, genes that are not sex‐limited in transmission or expression can be subjected to sex‐specific selection, constraining independent evolution across the sexes (Rice & Chippindale, 2002). This problem has been illustrated using human hip width as a possible example (Rice & Chippindale, 2001). The argument proposes that selection favors narrower hips for locomotion, but women are under additional selection for wider hips to facilitate childbirth. Thus, the frequency of genes affecting hip width fluctuates depending on their sex‐specific effects, with alleles for wider hips favored when expressed in women and disfavored when expressed in men (Rice & Chippindale, 2001). This is a putative example of intralocus sexual conflict, where alleles at a particular locus can have positive effects on fitness when expressed in one sex, but negative fitness effects when expressed in the other (Rice & Chippindale, 2001), thus preventing the independent evolution of the sexes toward their sex‐specific optimal character values and reducing fitness in one or both sexes (Arnqvist & Rowe, 2005; Bonduriansky & Chenoweth, 2009; Rice, 1996).

The sexually antagonistic selection that generates intralocus sexual conflict is widespread in natural populations (e.g., Cox & Calsbeek, 2009; Mainguy, Cote, Festa‐Bianchet, & Coltman, 2009) and has been detected in insects (e.g., Archer, Zajitschek, Sakaluk, Royle, & Hunt, 2012; Harano, Okada, Nakayama, Miyatake, & Hosken, 2010; Lewis, Wedell, & Hunt, 2011; Pischedda & Chippindale, 2006), vertebrates (Mokkonen et al., 2011), and plants (Delph et al., 2011). In addition to being taxonomically widespread, intralocus conflict has important and far‐reaching evolutionary effects, influencing demography (e.g., Berger et al., 2016; Katsuki, Harano, Miyatake, Okada, & Hosken, 2012), adaptation (e.g., Hawkes et al., 2016; Rostant, Kay, Wedell, & Hosken, 2015), life‐history strategies (e.g., Archer et al., 2015; Duxbury, Rostant, & Chapman, 2017), sex‐chromosome evolution (Mank et al., 2014), and speciation (Rice & Chippindale, 2002). Given the ubiquity of intralocus sexual conflict and its broad evolutionary impacts (reviewed in Bonduriansky & Chenoweth, 2009; van Doorn, 2009), it is important that we can accurately detect it and quantify its strength.

A common way to test for and quantify intralocus conflict is to calculate the intersexual genetic correlation for fitness (*r*_mf_), (e.g., Archer, Sakaluk, Selman, Royle, & Hunt, 2013; Brommer, Kirkpatrick, Qvarnström, & Gustafsson, 2007; Chippindale, Gibson, & Rice, 2001; Duffy, Joag, Radwan, Wedell, & Hosken, 2014; Pischedda & Chippindale, 2006; Punzalan, Delcourt, & Rundle, 2014; Collet et al., 2016). This correlation measures how similar genetic effects are when they are expressed in females or males (Bonduriansky & Chenoweth, 2009). A *r*_mf_ = −1 indicates that genes conferring high fitness in one sex confer low fitness in the other (i.e., strong intralocus sexual conflict) and that the sexes are not free to evolve to sex‐specific fitness optima. Conversely, a *r*_mf_ = 1 indicates that alleles that confer high fitness in one sex also confer high fitness in the other, illustrating the absence of intralocus conflict. While the *r*_mf_ is a powerful tool to quantify intralocus sexual conflict, correlations can be hard to interpret and make assumptions that are not always met (discussed in Bonduriansky & Chenoweth, 2009). Here, we show an additional complication with *r*_mf_ values—even in the absence of sexual conflict, the intracellular parasite *Wolbachia* can cause negative *r*_mf_ values for fitness, which suggests intralocus conflict but actually results from the action of non‐self genes.

*Wolbachia* is a vertically maternally transmitted cytoplasmic parasite found in a wide range of arthropods and filarial nematodes (Werren, 1997; Werren, Baldo, & Clark, 2008). It is estimated that over 60% of these taxa are infected, with most groups having intermediate frequencies of infection (i.e., not all individuals are infected: Hilgenboecker, Hammerstein, Schlattmann, Telschow, & Werren, 2008). *Wolbachia* can have complex effects on host physiology and reproduction, and one of the most common of these is the generation of unidirectional cytoplasmic incompatibility (CI) (Werren, 1997). When unidirectional CI occurs, uninfected females suffer from low fitness because sperm from males infected with *Wolbachia* are not able to produce functional zygotes with females that do not host the same *Wolbachia* strain. This means that matings between infected males and uninfected females produce no, or a very few, offspring. Infected females, however, have normal (or even higher—Weeks, Turelli, Harcombe, Reynolds, & Hoffmann, 2007) productivity regardless of their mate's infection status (Werren et al., 2008). In males, infection can reduce male sexual fitness components (Price & Wedell, 2008; Wedell, 2013; but also see, e.g., Okayama, Katsuki, Sumida, & Okada, 2016). As a result, infection can potentially impact fitness estimates in one or both sexes depending on the prevalence of infection. In principle, uninfected genotypes could have low female fitness because of CI, but high male fitness because CI is unidirectional and infected males are of lower fitness. Reduced fitness in one sex with corresponding high measures in the other sex could falsely suggest intralocus conflict—remembering this conflict by definition only applies to genes that are not sex‐limited in transmission or expression (Rice & Chippindale, 2002), which is not the case for *Wolbachia*.

Here, we tested whether CI caused by *Wolbachia* can increase the likelihood of detecting apparent intralocus sexual conflict (i.e., of falsely diagnosing intralocus conflict). We first simulate data to illustrate the rationale underlying this problem and then present empirical data on *Drosophila simulans* isofemale lines showing how CI can affect estimates of intralocus conflict. Given that *Wolbachia* is one of the most widespread endosymbionts in insect populations but wild populations contain a mix of infected and uninfected individuals (Hilgenboecker et al., 2008), its presence could potentially bias estimates of the prevalence of intralocus sexual conflict.

## 2 METHODS

### 2.1 Simulations

To test whether CI‐inducing *Wolbachia* could theoretically mirror intralocus sexual conflict, we simulated a series of datasets based on assumptions about the strength of CI and its effects on male fitness—we assume (a) no CI or that either 10% or 20% of genotypes experience CI, and random male fitness for uninfected males, or (b) no CI then 10% and 20% of genotypes experience CI, with uninfected males having higher than average fitness. This latter assumption is based on extensive data showing that male sexual fitness is frequently compromised in infected males (Champion de Crespigny & Wedell, 2006; Champion de Crespigny, Pitt, & Wedell, 2006; Price & Wedell, 2008; Snook, Clelend, Wolfner, & Karr, 2000; Wedell, 2013: but see, e.g., Okayama et al., 2016). The general structure of each dataset is the same: data for reproductive success (our proxy of fitness) were generated for 10 genotypes in males and females, creating a single mean fitness estimate for males and for females for each genotype. These data are replicated 100 times for each of set of assumptions (i.e., 100 estimates of male fitness, 100 estimates of female fitness/simulated dataset).

We initially assumed that male fitness and female fitness were completely uncorrelated. To create these data, fitness estimates were generated at random using the “rnorm” function in *R* (version 3.4.1) (R Core Team, 2017) around a mean of 60, and with a standard deviation of 15. We then simulated datasets with CI in 10% of genotypes. To do this, male fitness estimates were generated as described for our initial population but for females, fitness was reduced in one of the ten simulated genotypes—these female fitness estimates were generated at random, with a mean of 5 (±2*SD*). This means that unidirectional CI has reduced female fitness dramatically but not affected male fitness. To simulate 20% CI, we used the same approach to simulate CI in two of the ten genotypes. Finally, we simulated a possible scenario where uninfected males were of high sexual quality. This seems feasible given that parasite infection typically has some negative impact on the host (e.g., Schmid‐Hempel, 1998), and in the specific case of *Wolbachia* infection, infected males are frequently poor sexual competitors (Champion de Crespigny & Wedell, 2006; Price & Wedell, 2008; Wedell, 2013). Here, female data were generated as described above to generate CI in 10% or 20% of genotypes, but this time for uninfected genotypes (those with simulated CI), male fitness was increased (mean = 85, *SD* = 15).

To test how the introduction of CI affected our estimates of intersexual genetic correlations in each of these simulated fitness scenarios, Pearson's correlation coefficients (*r*_mf_) were calculated between male and female fitness estimates using the package “*psych*” and the function “*corr.test*” (Revelle, 2018). We regressed male and female fitness estimates by genotype in each of the 100 replicates/CI scenario (no CI, 10%‐CI, 20%‐CI) and random male fitness for uninfected males, and CI (with either 10% or 20% of genotypes uninfected) and high male fitness for uninfected males to generate 100 slope estimates/scenario. We calculated how many of these correlation estimates were positive or negative and how many were significant (*p* < 0.05), before testing whether the number of negative slopes differed across CI scenarios.

### 2.2 Testing how CI affects intersexual genetic correlations in inbred lines of *Drosophila simulans*

#### 2.2.1 Study species and animal maintenance

In April 2010, approximately 100 wild‐type *Drosophila simulans* females were collected from Athens, Greece, and used to establish isogenic female lines (hereafter isolines) at the University of Exeter, Cornwall, UK. These lines were maintained via full‐sib matings with non‐overlapping generations for 45 generations prior to beginning this experiment. After such prolonged inbreeding, each isoline is effectively genetically identical and can be considered an individual genotype (David et al., 2005). Thus, they can be used to test for genetic correlations (David et al., 2005) because they capture natural linkage, but other approaches (half‐sib designs) are probably better to precisely estimate specific genetic parameters when lines have been maintained for more than about five generations (Hoffmann & Parsons, 1988). Thus, we do not estimate genetic parameters per se here, but merely use the lines to estimate male–female correlations across genotypes (see below). Isolines were kept in 150‐ml (48 × 116 mm) vials with 30 ml of a cornmeal‐based food (Applied Scientific UK) and housed at 25°C with 50% relative humidity and with a 12:12‐hr light–dark cycle. Focal experimental animals came from these isolines, and the isolines which were used in this study were chosen randomly.

An outbred population cage was also established after the first generation of full‐sibling mating by adding flies from all isolines, and this was maintained at a size of ca. 800 individuals with overlapping generations and free mate choice. The population cage was housed in the laboratory at an ambient temperature of between 23 and 25°C and supplied with excess food. Males from this outbred population were used to assay the fitness of focal experimental females.

To assay the fitness of focal male flies, we used flies expressing the *ebony* mutation; *ebony* is a recessive allele that affects body pigmentation (Ashburner, Golic, & Hawley, 2005), providing a phenotypic marker that allows paternity to be assigned to wild‐type or *ebony* sires (Delcourt, Blows, & Rundle, 2009; Duffy et al., 2014). The *ebony* flies were supplied by the Tucson stock center and maintained in a large population cage (ca. 500–700 individuals) in the ambient laboratory environment (as above) with ad libitum food, free mate choice, and overlapping generations for four years prior to the beginning of the experiment.

### 2.3 Fitness assays

We measured fitness in each of 10 individuals of each sex, from each of 27 isolines, in three blocks (total = 30 flies/sex/line). Each block was assayed one generation (i.e., 12–14 days) apart. Flies were collected as virgins and then housed individually until sexually mature (3–4 days posteclosion) (Ashburner et al., 2005) after which they were assayed as described below.

We measured female fitness as the total number of adult offspring produced by a female over seven days, while being housed with two outbred males (see Duffy et al., 2014; and note housing females with multiple males increases female fitness: Taylor, Wigmore, Hodgson, Wedell, & Hosken, 2008). This measure of fitness captures female fecundity and egg to adult survival of offspring in competition with their siblings (Delcourt et al., 2009; Rundle, Chenoweth, & Blows, 2006). Male tester flies were collected as virgins at the same times as experimental females (housed at *n* = 6 flies/vial [60 ml])—ensuring all flies were 3–4 days old on the first assay day. These triads of flies were housed in 35‐ml vials with 10 ml of food for 48 hr, before being moved to a second vial for a further 48 hr, and then moved to a final vial for 72 hr. After this, all adults were removed from these vials. Throughout, flies were transferred without anesthesia via aspiration in order to avoid any effects on female oviposition behavior or fertility associated with CO_2_ anesthesia (Champion De Crespigny & Wedell, 2008). Eggs and larvae from each of these three vials were allowed to develop. After six days, vials were monitored daily for eclosion, and once the first eclosion from a vial was observed, vials were incubated for a further eight days, and then all offspring that had emerged as adults were counted. This ensured that only the offspring of focal females were scored, rather than their grand‐offspring (Sharma, Mitchell, Hunt, Tregenza, & Hosken, 2012; Sharma, Tregenza, & Hosken, 2010; Taylor et al., 2008).

We measured male fitness as reproductive success when competing with other conspecific males. This measure of male fitness has been used in other studies (Chippindale et al., 2001; Delcourt et al., 2009; Duffy et al., 2014; Mills, Koskela, & Mappes, 2011) and captures several components of fitness including male mating success, male fertility, and the survival to emergence of male offspring when in direct competition with offspring sired by the *ebony* competitors (Delcourt et al., 2009). To assay male reproductive success, we housed a single sexually mature, virgin male in a 35‐ml vial with 10 ml of food and four sexually mature virgin competitor males (i.e., tester males) exhibiting the *ebony* mutation. The isoline and *ebony* males competed for fertilizations with one, sexually mature, virgin *ebony* female (note that housing female *D. simulans* with multiple males does not have the negative fitness effects seen in *D. melanogaster*: e.g., Taylor et al., 2008). As with the female assay, experimental and tester flies were collected as virgins at the same times to ensure all flies were the same age (3–4 days old) on the day that fitness was assayed. These cohorts of flies were housed in three different vials, as described for the female fitness assay. Offspring were counted from each of the three vials as described above and scored as being wild‐type (and so sired by the focal isoline male) or *ebony* (and therefore sired by a competing male). We calculated the fitness of an isoline male by scoring the ratio of wild‐type:*ebony* offspring in each vial.

### 2.4 *Wolbachia* screening

*Wolbachia* infection status was determined via PCR analysis in 15 females from each of five isolines (see Table 2). Females were assayed from the two female isolines that showed the most pronounced sex differences in fitness (dotted lines Figure 3; F7, F12). The remaining three lines had either very high or intermediate female fitness values (see Table 2 for more information on mean female fertility in these lines).

To assay *Wolbachia* status, individual adult females from each line were squashed in 25 mM NaCl, 10 mM Tris‐HCl pH¼ 8.0, 1 mM EDTA, 200 lg/ml proteinase K and incubated for 30 min at 37–8°C. Following incubation, proteinase K was inactivated at 95°C for 5 min. The supernatant was then directly used for PCR amplification under the following thermal profile: 94°C for 4 min then 94°C for 1 min, 52°C for 1 min and 72°C for 1 min per cycle for 35 cycles and finally 72°C for 4 min. Ten microliters of each sample was electrophoresed on 1% agarose gels, stained with ethidium bromide, and visualized under ultraviolet illumination. We used the *wsp* (*Wolbachia* surface protein) primers *wsp* 81F (59‐TGGTCCAATAAGTGATGAAGAAAC‐39) and *wsp* 691R (59‐AAAAATTAAACGCTACTCCA‐39), which amplify an approximately 600‐bp fragment of the *wsp* gene in *Wolbachia* strains which infect *D. simulans* (Teixeira, Ferreira, & Ashburner, 2008; Zhou, Rousset, & O'Neill, 1998). *Wsp* is a single‐copy gene coding for an outer membrane protein of *Wolbachia* (Braig, Zhou, Dobson, & O'Neill, 1998) and has been widely used for *Wolbachia* screening in *Drosophila* (Dobson et al., 1999; Jeyaprakash & Hoy, 2000; Müller, Mühlen, & Valiati, & Valente, 2012; Van Meer, Witteveldt, & Stouthamer, 1999). We did not assess the infection status of tester males as they came from stock populations, and hence, their precise status could not be replicated, and the individual males used had been discarded before any evidence of CI was recorded. However, subsequent tests indicate that these populations (wild‐type and *ebony*) are infected like many *Drosophila* stocks ((Clark, Anderson, Cande & Karr, 2005).

### 2.5 MLST sequence typing

To test whether the *Wolbachia* strain in the isolines shared sequence types with a CI‐inducing *Wolbachia* strain (Dsim_A_wRI (id:11)), we used multi‐locus sequence typing (MLST) of five conserved genes (*gatB*,* coxA*,* hcpA*,* ftsZ,* and *fbpA*) and four WSP hypervariable regions (HVRs) performed in line with standard protocols (for details, please see https://pubmlst.org/wolbachia/info/protocols.shtml).

### 2.6 Curing flies of *Wolbachia*

To examine intersexual fitness correlations without the confounding effects of *Wolbachia,* we cured replicate isolines of the bacteria. This was performed 12 generations after finishing the final round of fitness assays described above. Here, we established replicates (*n* = 25/sex/line) of all 27 of the isofemale lines using during the initial experiment. Additionally, large subsets of flies from the *ebony* and the outbred population cages (used for male and female assays) were established. We used the wide spectrum antibiotic tetracycline HCL (Sigma‐Aldrich) to remove *Wolbachia* as outlined in Hoffmann, Turelli, and Simmons (1986). Briefly, after cooling food media to 48°C, per 40 ml of food media per 150 ml isoline vial we added 1 ml of a 12.3 mg/ml tetracycline solution providing a final concentration of ca. 0.3 mg/ml (0.03%). Tetracycline was added to the food media of the replicate isolines and the subset populations established from the *ebony* and outbred population cages, such that these populations were also cured of *Wolbachia* at the same time as isolines were cured. This was performed for three generations for all fly populations, after which the presence or absence of *Wolbachia* was tested with PCR analysis of 15 females per isoline (using the same protocol as described above). PCR analyses showed that all isolines (*n* = 27) were *Wolbachia‐*free. Following confirmation of *Wolbachia* absence, we allowed for three generation of “recovery” on non‐tetracycline‐treated media before repeating fitness assays (as described above).

### 2.7 Statistics

Prior to analyses, data were *Z‐*transformed—using the mean and standard deviation for each sex within each block—so that male fitness and female fitness were on comparable scales. In the initial fitness assays (i.e., before CI was identified or lines cured of *Wolbachia*), one outlier was identified and removed. Means for each line were then calculated and used to determine Pearson's correlation coefficients (*r*_mf_) between male and female fitness. To examine intersexual fitness correlations among isolines, we used a linear mixed‐effects model (“lme4” package—Bates, Maechler, Bolker, & Walker, 2015) in R version 3.4.3 (R Core Team, 2017). Sex and isoline were fit as fixed effects with block included as a random effect. *p* Values for the fixed effects were calculated using *F* statistics in the “LmerTest” package (Devigili et al., 2018; Kuznetsova, Brockhoff, & Christensen, 2015) with denominator degrees of freedom generated using Satterthwaite's approximation in the “ANOVA” function from the same R package. *p* Values for the random effects were calculated based on likelihood ratio chi‐square tests.

When we identified lines that appeared to have CI, we reanalyzed our data. To do this, we removed the two affected lines and then re‐standardized the data (i.e., new Z‐transformed line averages were calculated), before repeating the analyses described above. To compare correlation coefficients for these datasets (i.e., including and excluding lines with CI), we used the following equation:

$$t=\frac{b_{1}-b_{2}}{\sqrt{Sb_{1}^{2}+Sb_{2}^{2}}}$$

where *b*_1_ and *b*_2_ are the slopes to be compared and s is the standard error associated with each slope estimate (Zar, 1999).

## 3 RESULTS

### 3.1 Simulated data

**Figure 1:**
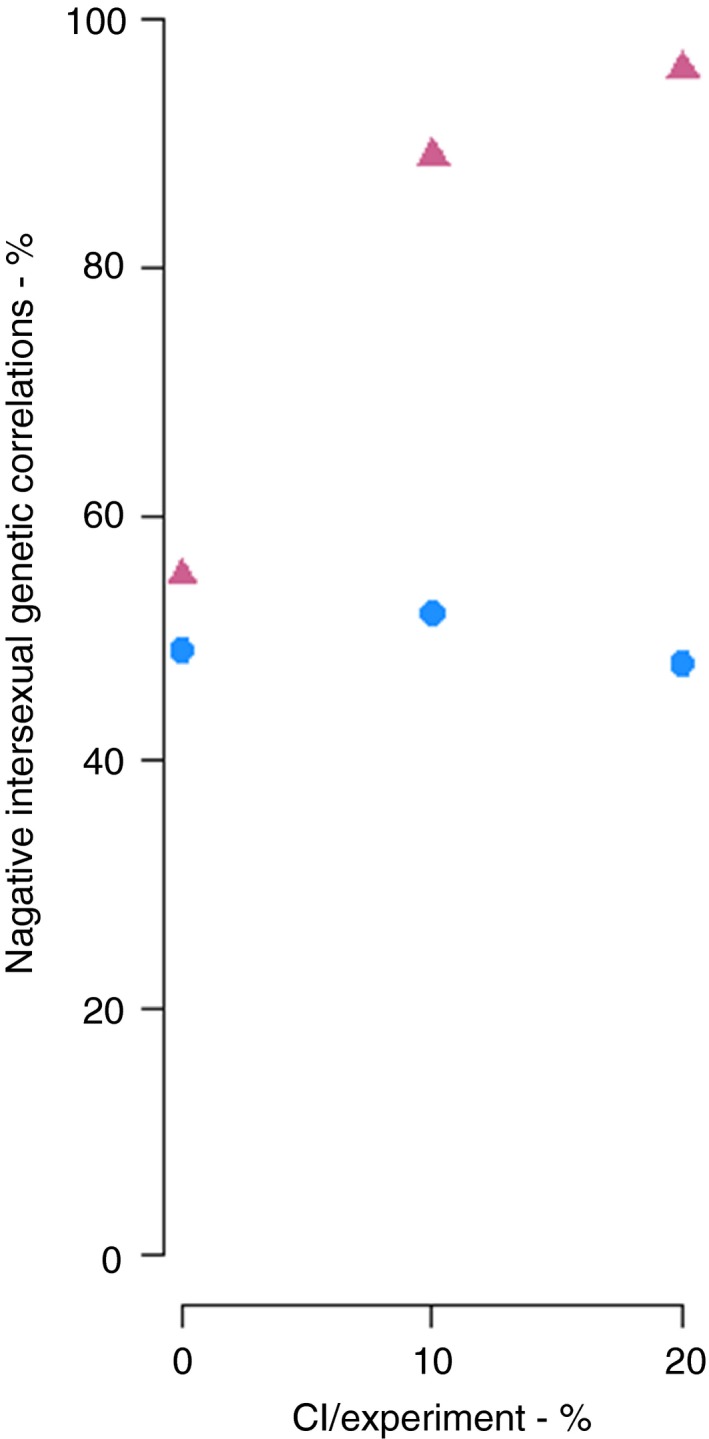
The percentage of intersexual genetic correlations that were negative when 0%, 10%, and 20% of genotypes were uninfected with *Wolbachia* (i.e., cytoplasmic incompatibility (CI) would have been seen in 0%, 10%, 20% of crosses between genotypes) in a simulated dataset. Blue dots show the situation when relationship between male and female fitness was randomized. Red triangles show the situation when CI causes uninfected females to have low fitness, but uninfected males have high fitness—a common situation in nature. Note that in this latter case, uneven infection, which would result in CI, almost always results in negative male–female fitness associations

When the relationship between male and female fitness was generated at random and there was no CI, ca. 50% of correlations were positive and 50% were negative, with only 5 of the 100 correlations being significant. This is precisely what we would expect. When we simulated CI at random in 10 or 20% of genotypes, such that uninfected genotypes have low female fitness because of CI, but males with high, medium, or low fitness estimates nothing really changed (Figure 1 and Supporting Information Figure S1—blue points). However, if the uninfected genotypes include males with high relative fitness (i.e., males without *Wolbachia* infection tend to be more fertile than infected males), but CI causes a reduction in uninfected female fitness, even modest levels of CI can create significant, negative correlations for fitness, which mirrors expectations under intralocus conflict (Figure 1 and Supporting Information Figure S1—pink points). The reason for this is illustrated in Supporting Information Figure S2—when male fitness is high, unidirectional CI mirrors intralocus conflict in these genotypes, skewing overall correlation estimates. If we consider just the significant slopes (i.e., simulations that produced statistically significant regressions), we note that with CI and non‐infected males having higher fitness, *all slopes were negative*, while in all other simulation scenarios, zero or positive slopes were detected (Table 1). Contingency table testing reveals these associations are not independent, either when tested across all combinations (*χ* ^2^ = 18.1; *df* = 5; *p* < 0.003) or when tested only when uninfected male fitness is high (*χ* ^2^ = 17.2; *df* = 2; *p* < 0.001). Additionally, the number of significant negative versus positive values for CI simulations when uninfected males had high fitness and 10% (18/0) or 20% (31/0) of genotypes were uninfected (Table 1) are both highly significant in binomial tests (BinP = 0.5) tests (all *z* > 2.84; all *p* < 0.001). This indicates that CI in uninfected females plus higher fitness in uninfected males together generates significantly more negative fitness associations than expected by chance.

**Table 1:** The numbers of significant positive and negative intersexual fitness correlations over the range of cytoplasmic incompatibility and relative male fitness parameters we simulated

CI %	Relative male fitness	Number of positive correlations	Number of negative correlations	Number of significant positive correlations	Number of significant negative correlations
0	Random	51	49	3	2
10	Random	48	52	2	4
20	Random	52	48	1	3
0	High	45	55	2	3
10	High	11	89	0	18
20	High	4	96	0	31

Note

CI % is the proportion of genotypes that were not infected with *Wolbachia*, while random male fitness meant uninfected males could take on any fitness value (low, medium, high) and high male fitness meant uninfected males were on average better sexual competitors. As can be seen, with high male fitness and 10% or more CI, negative fitness associations become the norm.

### 3.2 *Drosophila simulans* isolines

**Figure 2:**
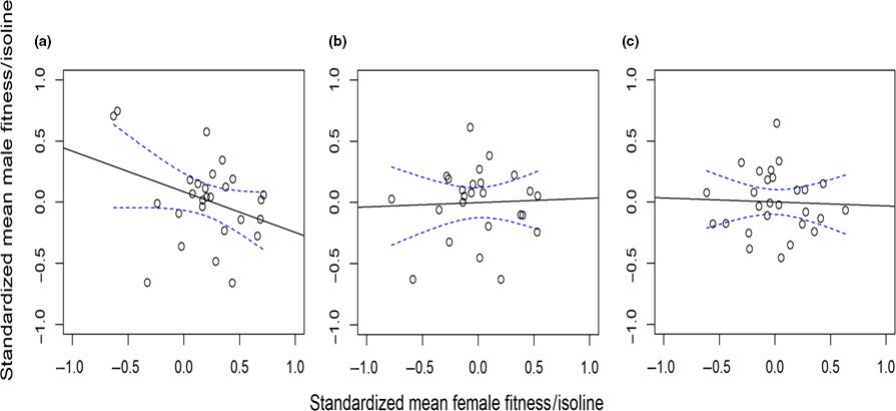
(a–c) Empirical data generated from *Drosophila simulans* isolines. Plot a shows the intersexual fitness correlation using data from all assayed lines (*n* = 27); the negative association is non‐significant, but only marginally so (*t* = −1.801, *df* = 25, *r*_mf_ = −0.34, *p = *0.08). Plot b depicts empirical data generated from *Drosophila simulans* isolines omitting the two uninfected isolines from the analysis (*n* = 25). The sign of the intersexual correlation changes from negative to flat (*t* = 0.196, *df* = 23, *r*_mf_ = 0.04, *p = *0.85). Plot c depicts a similar outcome when analyzing results from flies that had all been cured of *Wolbachia* infection (*t* = −0.193, *df* = 25, *r*_mf_ = −0.038, *p* = 0.84). Blue lines represent 95% confidence envelopes

**Figure 3:**
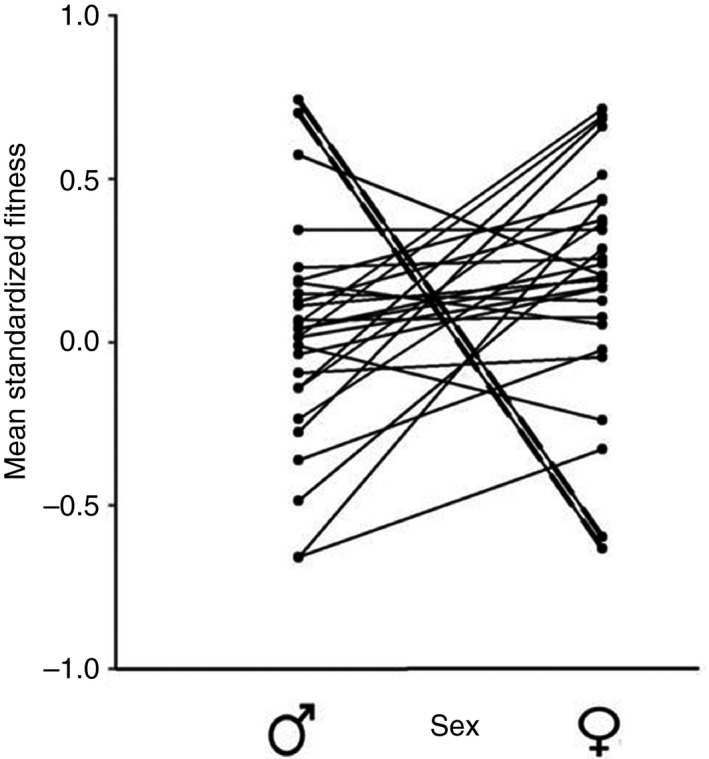
Interaction plot for the significant sex‐by‐isoline interaction (*F*_1,1582_ = 10.72, *p* < 0.001). The bold, dashed lines depict the top two highest male fitness lines, which are the corresponding lowest female fitness lines. These isolines were subsequently found to be uninfected with *Wolbachia,* which probably resulted in cytoplasmic incompatibility fitness reductions for the females

There was a negative, marginally non‐significant *r*_mf_ (Figure 2a) among isolines (genotypes) (*t* = −1.801, *df* = 25, *r*_mf_ = −0.34, *p = *0.08). The mixed‐effects model indicated a significant sex‐by‐isoline interaction (*F*_1,1582_ = 10.72, *p* < 0.001); this significant interaction term including isoline indicates the presence of genetic variation between lines. However, the main effects of sex (*F*_1,1582_ = 1.17, *p* 0.28) and isoline (*F*_1,1582_ = 3.04, *p = *0.081) were not significant. The random effect of block was significant ($\chi_{1}^{2}$ = 27.0, *p* = 0.01). Females from two of the isolines produced very few offspring, although males from these lines had particularly good fertility (Figure 3). These isolines also failed to produce offspring in a second experiment in the laboratory (data not shown), suggestive of unidirectional CI driven by *Wolbachia*. The *Wolbachia* infection status of these lines, and three others (see Table 2), was determined using PCR analysis. Results indicated that both lines that failed to produce offspring were uninfected with *Wolbachia* but the other three isolines had relatively high levels of infection (Table 2). MLST sequencing showed that infected isolines carry a CI‐inducing *Wolbachia* strain.

**Table 2:** Results from *Wolbachia* screening using PCR analyses of the two highest and two lowest female fitness isolines and one randomly chosen line (F72)

Isoline ID	% Infected (*n*)	Mean fertility (±*SE*)	Fitness rank in fertility assay (1 = highest, 27 = lowest)
F53	80 (12/15)	71.7 (7.3)	1
F8	93 (14/15)	71.1 (6.6)	2
F72	93 (14/15)	65.4 (8.7)	6
F12	0.0 (0/15)	29.2 (4.5)	26
F7	0.0 (0/15)	28.5 (5.0)	27

Note

Fifteen females were sampled from each line. All of the flies from the two lowest female fitness lines were uninfected. Infection rates were over 80% for all other lines. Fertility values and fitness ranks correspond to the initial fitness assays of all 27 isolines. The line with the greatest fitness (highest average female fecundity) has a rank of 1, and the line with the lowest fitness has a rank of 27.

To test whether the low female fitness values for these lines influenced the estimated intersexual genetic correlation for fitness (*r*_mf_), an additional correlation analysis was performed without the uninfected lines (i.e., *n* = 25 lines). Prior to analysis, data were again standardized but excluding data from the uninfected isolines. The *r*_mf_ was non‐significant but the slope changed from negative to flat (*t* = 0.196, *df* = 23, *r*_mf_ = 0.04, *p = *0.85) (Figure 2b). Comparing these slopes revealed that the difference between them was marginally non‐significant (*t*_(1)_ = −1.14, *df* = 48, *p = *0.08). Once all populations were cured of *Wolbachia*, we repeated the fitness assays and calculated new *r*_mf_ estimates for fitness and found no indication of a negative intersexual fitness correlation (*t* = −0.193, *df* = 25, *r*_mf_ = −0.038, *p* = 0.84) (Figure 2c).

## 4 DISCUSSION

Intralocus sexual conflict is important and likely to be pervasive (Bonduriansky & Chenoweth, 2009; Mank et al., 2014; Pennell & Morrow, 2013). It occurs when alleles that are not sex‐limited in their expression or transmission encode traits that have different optimal values in either sex. Sex‐specific patterns of selection then lead to an intersexual evolutionary tug‐of‐war over allelic values at specific loci (Rice & Chippindale, 2002). Here, we illustrate a potential problem with detection of intralocus conflict in insects using a combination of simulated and experimental data. Unidirectional CI caused by crosses between *Wolbachia‐*infected males and *Wolbachia*‐uninfected females can create significant negative intersexual genetic correlations for fitness, indicative of intralocus conflict, even when there are no sexually antagonistic alleles *sensu* *stricto* segregating in a population. Note that this parasitic endosymbiont is maternally transmitted, and so even if we broaden the definition of intralocus conflict to include non‐self genes, we are still dealing with a different phenomenon (i.e., because transmission is sex‐biased: see definition in Rice & Chippindale, 2002). The interplay between genomic parasites and intralocus conflict, and the consequences of it, has been discussed at length elsewhere (Mank et al., 2014). Here, we merely illustrate how CI can confound conflict estimates using simulated data, and then present data that broadly support the potential problem in *D. simulans*. If CI can mimic, or even just strengthen estimates of sexual conflict, this has implications for tests of intralocus conflict in insects when the *Wolbachia* infection status of experimental populations is unknown.

The argument is straightforward—if sexual fitness components are used as a measure of fitness and if some genotypes are not infected with *Wolbachia*, but some are (as seems to be the usual case in nature: Hilgenboecker et al., 2008), this can lead to unidirectional CI and lower fitness of infected males, and hence comparisons of male and female fitness mimic intralocus conflict. This is because when uninfected females are mated with infected males, they will produce few offspring (Werren, 1997; Werren et al., 2008), but uninfected males may have higher fitness because infected males tend to be poorer sexually (e.g., Champion de Crespigny et al, 2006; Wedell, 2013). However, *Wolbachia‐*infected females will produce viable offspring regardless of paternal infection status (Werren, 1997; Werren et al., 2008). This means that infected females are good but infected males can be poor, and uninfected females are poor but uninfected males are good, potentially generating negative intersexual fitness correlations that mimic conflict, as our simple simulated data show. It is worth noting that our simulations may have overestimated the sex‐specific fitness costs of *Wolbachia*—as shown by our empirical data*—*because effects will depend on infection prevalence and penetrance of CI. Furthermore, the strength of CI effects and male fitness impacts can vary across taxa (e.g., Okayama et al., 2016). However, the point remains that *Wolbachia‐*driven CI can in principle mimic the effects of intralocus sexual conflict.

Simulation outcomes were broadly supported by the empirical data*.* In *D. simulans* isofemale lines, we found evidence of a negative intersexual fitness correlation—a strong but non‐significant trend. However, this signal of sexual conflict was largely driven by two isolines where males had high fitness and females had low fitness. Data were consistent with this resulting from CI — curing flies of infection eliminated the negative trend. While significant crossing over of fitness ranks remained even without these two lines, the sign of the association went from negative to weakly positive when they were excluded from the analysis. It is important to note that these two lines did not have significant effects on our results—the negative *r*_mf_ for fitness was non‐significant even when these lines were included in analyses. However, what is clear from these data is that males from lines with CI had the highest fertility estimates of all males, showing that our second simulation scenario is biologically feasible (i.e., where CI is associated with low female fitness but high male fitness estimates) and the presence of CI in just two lines out of 27 turned a flat and non‐significant *r*_mf_ for fitness into a negative, very nearly significant correlation. This illustrates the potential for CI to strengthen (or create) signals of intralocus sexual conflict. It must be noted again that in our empirical data, fitness regressions were not significant, which implies the CI and fitness effects in the flies were weaker than in our simulations. This may be because CI effects could be reduced by mate choice for example. However, genetic correlations, which our isoline regressions represent (David et al., 2005), usually have very large errors associated with them (Lynch & Walsh, 1998) making statistical significance hard to achieve (e.g., see figure 3 in Sharma, Wilson, & Hosken, 2016). This is why in other contexts it has been recommended that correlation magnitudes are reported regardless of statistical significance (Sharma et al., 2016).

It is important to note that we cannot say definitively that CI drove the empirical results we observe because the *Wolbachia* infection status of the tester males used in this experiment could not be confirmed—tester males were not retained following mating assays. However, the strong negative correlations for fitness seen in the isolines that showed signals of CI disappeared in assays with flies cured of *Wolbachia* infection, and hence, there was no longer any presence of CI. Additionally, *D. simulans* displays much higher levels of CI than closely related species such as *D. melanogaster* (Champion de Crespigny et al, 2006; Hoffmann, Clancy, & Merton, 1994; Hoffmann, Hercus, & Dagher, 1998; Solignac, Vautrin, & Rousset, 1994), and levels of CI can approach 100% meaning that mating between infected and uninfected flies does not produce any offspring at all (Callaini, Riparbelli, & Giordano, 1996; Champion de Crespigny, Hurst, & Wedell, 2008; Lassy & Karr, 1996), which is in keeping with our results. Furthermore, CI will help *Wolbachia* spread to at least intermediate frequencies in population cages, making it likely that our male testers (or many of them) were infected, all the more so given that most of the isolines we tested were infected and prevalence in these lines was very high (≥80%). So the empirical data show the precise pattern expected if CI was affecting fitness associations. In any case, our simulated data show how easily CI could create significant negative *r*_mf_ for fitness, so our main point remains unchanged.

Given the potential for *Wolbachia* to influence fitness estimates, and potentially to create signals of intralocus conflict, what should we do about it? Reproductive success is the primary sex‐specific measure of fitness (e.g., Baker et al., 2001; Nguyen & Moehring, 2017; Punzalan et al., 2014; Potdar, Daniel, Thomas, Lall, & Sheeba, 2018; Sharp & Agrawal, 2008; Tobler, Hermisson, & Schlötterer, 2015; Travers, Garcia‐Gonzalez, & Simmons, 2016), but in a range of invertebrates, sexual fitness might be affected by *Wolbachia*. This is particularly true for *Drosophila* species given that in 2005 it was reported that ca. 30% of stocks at the Bloomington *Drosophila* stock center were infected with *Wolbachia* (Clark et al., 2005). Furthermore, some frequently used stocks such as the *Drosophila* Genetic Reference Panel (MacKay et al., 2012) are naturally infected with *Wolbachia* (ca. 50%) (Arbuthnott, Levin, & Promislow, 2016). Researchers may therefore be working with infected stocks*,* which in turn may be having an unknown effect on experimental outcomes (Clark et al., 2005). It is therefore important to screen for *Wolbachia* in insect studies testing for intralocus conflict. In addition to screening for the presence or absence of *Wolbachia,* it is also important to determine which strain is present because of incompatibilities between different strains (Rousset & de Stordeur, 1994; Rousset, Braig, & O'Neill, 1999; Werren et al., 2008).

An alternative argument, however, is that the negative fitness correlations across the sexes caused by intracellular parasites such as *Wolbachia* are an extension of sexual conflict. That is, sexual conflict is not only fought over alleles within male and female genomes, but also over any parasites that they may host. In this case, removing *Wolbachia* could lead to a biologically inaccurate assessment of the potential for sexual conflict, but if so, a redefining of intralocus conflict is needed and this would blur lines between sex‐specific parasitic effects and intralocus sexual conflict. As such, we prefer the current status quo and the need for caution with respect to infection status.

In summary, we provide evidence that infection with cytoplasmic incompatibility inducing *Wolbachia* can potentially generate the negative intersexual fitness correlations that are used as the definitive signature of intralocus sexual conflict even when intralocus conflict may be absent or minor. We therefore suggest that documenting the infection status of insect test subjects is needed before conclusions about the causes of male–female fitness associations are drawn.

## CONFLICT OF INTERESTS

There are no competing interests.

## AUTHOR CONTRIBUTIONS

ED, MS, RJ, JR, NW, and DJH conceived the experiments. RA and DJH conceived the simulations. ED, MS, RJ, and MP conducted the experiments. RA conducted the simulations. ED, RA, and DJH analyzed the data. ED, RA, and DJH wrote the first draft of the manuscript. All authors contributed to data interpretation, manuscript drafting, and approval of the final draft.

## DATA ACCESSIBILITY

Data will be archived in Dryad on manuscript publication.
