# Supplementary material for: Wolbachia infection can bias estimates of intralocus sexual conflict
Source: Ecol Evol. 2018 Dec 26;9(1):328–38. doi: 10.1002/ece3.4744 (PMC6342094; doi:10.1002/ece3.4744)
Supplement: Supplementary file 1 [file ECE3-9-328-s001.docx]

**WOLBACHIA INFECTION CAN BIAS ESTIMATES OF INTRALOCUS SEXUAL CONFLICT.**

**SUPPLEMENTARY INFORMATION**

**Supplementary figure 1:** The percentage of statistically significant intersexual genetic correlations that were negative when 0, 10 and 20% of genotypes were uninfected with *Wolbachia* (i.e. cytoplasmic incompatibility (CI) would have been seen in 0, 10, 20% of crosses between genotypes) in a simulated data-set. Blue dots show the situation when relationship between male and female fitness was randomised. Red-triangles show the situation when CI causes uninfected females to have low fitness, but uninfected males have high fitness – a situation that is common in nature. Note that in this latter case, uneven infection, which would result in CI, massively inflates the number of significant negative male-female fitness associations.


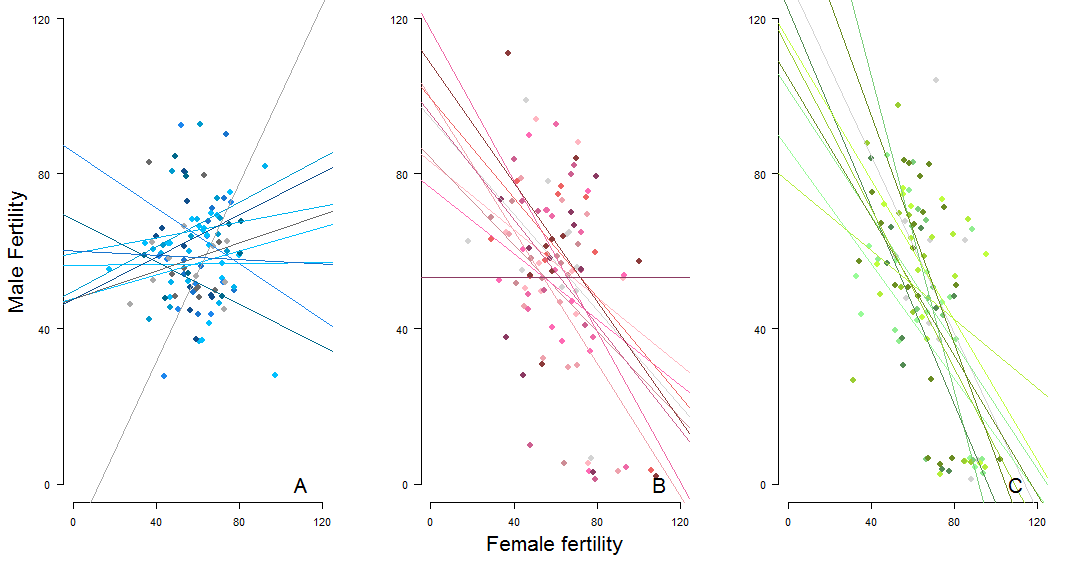


**Supplementary figure 2:** The percentage of significant negative genetic correlations when 0 (A), 10 (B) and 20% (C) of genotypes were uninfected with *Wolbachia* (i.e. cytoplasmic incompatibility (CI) would have been seen in 0, 10, 20% of crosses between genotypes). For the analyses, data were simulated for each of 10 genotypes 100 times (for each scenario) – however for the purposes of clarity in this figure, we simulated data for each of 10 genotypes, 10 times. Each point represents the population mean fertility estimates for males and females from each genotype, in each of the 10 simulations with different colours connecting data (and regression lines) from the same experiment. Lines are plotted using the function “abline” and linear regression models. For these data, in genotypes experiencing CI, females have low fitness (randomly generated from a normal distribution with mean of 5 and sd of 2) but males have high fitness estimates (randomly generated from a normal distribution with mean of 85 and sd of 15), while for genotypes without CI, male and female fitness are randomly generated numbers a normal distribution with a mean of 60 and sd of 15.
